# Quality of life of fitness professionals in Portugal: Comparative and correlation study

**DOI:** 10.3389/fpsyg.2022.958063

**Published:** 2022-08-24

**Authors:** Isabel Vieira, Dulce Esteves, Liliana Ramos, Vera Simões, Susana Franco

**Affiliations:** ^1^School of Sports of Rio Maior, Polytechnic Institute of Santarém, Rio Maior, Portugal; ^2^Quality of Life Research Center (CIEQV), Santarém, Portugal; ^3^Department of Sport Sciences, Faculty of Social and Human Sciences, University of Beira Interior, Covilhã, Portugal; ^4^Research Centre in Sports Sciences, Health Sciences and Human Development (CIDESD), Vila Real, Portugal

**Keywords:** fitness, fitness professionals, quality of life, WHOQOL-Bref, job characteristics

## Abstract

Fitness has been revealing a positive impact on the quality of life (QoL) of practitioners and fitness professionals (FPs) represent a role model for their customers in the fitness industry, emphasizing the need to know their QoL indices. The purpose of this study is to characterize the QoL of FPs in Portugal, compare it between groups and correlate QoL with sociodemographic and work-related variables. A total of 388 FPs answered an online survey about sociodemographic and work-related variables and the Portuguese version of the World Health Organization Bref QoL Assessment. Descriptive analysis, *t*-test, ANOVA, Kruskal-Wallis, Pearson and Spearman were used for statistical analysis. The results suggest that FPs in Portugal have different levels of QoL, considering the high standard deviation, with lower average indices than other countries. The lowest scores of QoL were verified in the environment domain. The results of the comparison between groups suggest that being male, married and having a master’s degree or higher were the characteristics with better indices of QoL. Age, professional experience and net salary reveal a positive relation/association with QoL. Body&mind group classes (GC) per week, maximal GC per day, paid and unpaid working hours per week and annual expenses related to the profession reveal a negative relation/association. Maximal GC per day results could be an important finding to help minimize the physical problems among FPs and body&mind GC per week results could be an indicator of the FPs adaptation that allows them to remain on the profession. Unpaid working hours per week has the highest number of significant relations/associations with QoL. These findings could be important to improve the QoL of FPs. Thus, they could result in better work capacity and, therefore, fewer professionals would abandon the profession. It would also have a positive impact on the fitness industry and on the promotion of physical activity for a healthier society. More research is needed regarding the QoL of FPs.

## Introduction

Quality of life (QoL) represents an important concept in health and medicine fields (Harper et al., 1998; Haraldstad et al., 2019). The World Health Organization (WHO) defines QoL “as individual’s perception of their position in life, in the context of the culture in which they live and in relation to their goals, expectations, standards, and concerns” (Kuyken and The WHOQOL Group, 1995, p. 1405; Harper et al., 1998, p. 551). Minayo et al. (2000) add that the notion of QoL also includes the idea of sustainable development. According to a preliminary reflection on the United Nations “New Agenda” (Okado and Quinelli, 2016), the first dimension of the global megatrends 2,030 and sustainable development goals point to a new population profile, who tend to require better public policies and higher QoL from governments.

According to the Ottawa Charter (World Health Ognanization [WHO], 1986), health is an important dimension of QoL and a considerable resource for social, economic, and personal development. Since QoL is an important measure of health impact, there is a recent interest in its measurement in public policies (Campos and Neto, 2008).

Physical activity plays an important role in a healthy lifestyle and fitness has been revealing a positive impact on the QoL of the practitioners (Puciato et al., 2017; Barranco-Ruiz et al., 2020; Soares-Miranda et al., 2021). This positive relation/association has been reported in general QoL and in different domains (physical, psychological, social relations, and environment), at different ages, in healthy populations or in a population with a problem or pathology. Fitness professionals (FPs), through their intervention, contribute to the improvement of the QoL of their customers. However, few studies have been found about the QoL of these professionals.

In 2019, the global fitness industry had approximately 184 million customers and nearly 210,000 gyms/health clubs around the world (International Health Racquet & Sportsclub Association [IHRSA], 2020). In 2020, the COVID-19 pandemic led to the permanent closure of 17% of clubs in the United States and 40–50% in other countries (International Health Racquet & Sportsclub Association [IHRSA]., 2021). In the European market alone 1.4% of the clubs were closed and 15.4% of memberships were canceled (compared to 2019) (Rutgers et al., 2021). According to the Fitness Barometer 2020 (Pedragosa and Cardadeio, 2021), there was a 22.9% decrease in the average number of memberships in the fitness sector in Portugal, 27% of fitness clubs closed down and there was an 18.7% decrease in the number of fitness instructors in the industry. In Portugal’s Fitness Barometer 2021 (Pedragosa et al., 2022), the decrease continues in the number of practitioners (491,355 in 2020 to 465,600 in 2021) and in the number of FPs working full-time in one gym or gym company (less 1.73% than in 2020). In 2021, there were 21,946 FPs titles in Portugal, 16,604 of which were Physical Exercise Technician (TEF) and 5,342 Technical Director (DT) (Instituto Português do Desporto e Juventude [IPDJ], 2022). Europe Active 2022 report (Rutgers et al., 2022) reveals a small recovery of the fitness industry in 2021, comparatively to 2020. However, the fitness industry is still struggling, considering the decline of 11.4% in revenues. In addition, this could also have implications on the QoL of FPs. On the other hand, QoL is associated with job satisfaction (Faragher et al., 2005; Bevilacqua et al., 2014; Neto et al., 2020), which is considered to be the most important factor that predicts turnover intention in many studies (Alam and Asim, 2019). Consequently, the information regarding the QoL of FPs could be helpful for the recovery of the fitness industry.

A pilot study conducted in Portugal (Vieira et al., 2019) reveals that FPs have positive perceptions about their QoL. The average score of Overall QoL and General Health (OQoLGH) was 79.4. The physical and social relations domains presented the highest scores (80.8 and 80.7, respectively), followed by the psychological (79.4) and the environment (73.5) domains. Despite these results, FPs are exposed to certain factors that may influence their QoL and some of them are related to the work context. According to Neto et al. (2020), QoL is associated with job satisfaction and ability to work. However, the routine of these professionals presents characteristics of an extensive workload and broad physical effort, which can interfere with their perception of QoL.

To Faragher et al. (2005), the consequences of lower levels of job satisfaction could interfere with physical and mental health, QoL, stress, self-esteem, and individual life. Marín-Farrona et al. (2021) suggest that better working conditions reduce the FPs response to stress.

A recent study on job satisfaction of FPs in Portugal (Ramos et al., 2021a) showed that lower degrees of job satisfaction were regarding salary, opportunities for promotion and stability at work. A characterization study of FPs in Portugal (Ramos et al., 2021b) reveals that 42% of FPs work 34 h per week (hours/week) or less in fitness and 37.7% reconcile the career in fitness with another job. Data from the Portuguese Fitness Barometer 2020 (Pedragosa and Cardadeio, 2021) shows that only 26% of FPs work full-time at one gym or gym company and 43% work less than 20 h/week. The number of FPs working full-time at one gym or gym company decreased from 9,822 in 2020 to 9,652 in 2021 (Pedragosa et al., 2022). In the characterization study of FPs in Portugal (Ramos et al., 2021b), it was found that most FPs are independent workers (68.1%) and the net salary received is between €631,98 and €842,63, despite that 86.2% had a bachelors’ degree or higher.^1^ The functions that are mostly performed are directly related to exercise. On the top of the list are the fitness group classes (GC) (82.02% of the FPs perform this function, with a weekly average of 8.66 h). This can represent a concern because many studies are highlighting the physical problems of FPs of GCs.

In 1988, FPs of GCs had twice the probability to be injured as the practitioners of those classes (Garrick et al., 1986). In 2001, the reported rate of injury among aerobic class FPs in Sydney (Australia) was 77% (du Toit and Smith, 2001). 15 years later, a survey of Zumba class dancers revealed that the odds of injury were seven times greater for registered instructors than for class practitioners (Domene et al., 2017). Recent research about FPs of GCs in Norway refers the need to limit the weekly number of GCs (instruction loading), especially for classes with high metabolic and/or mechanical loading (Bratland-Sanda et al., 2015). The instruction of exercise classes can be considered as an area of performance, which can be more physically and mentally demanding than participating in such classes (Bratland-Sanda et al., 2015).

There are also reports of other health problems related to this profession. The prevalence of urinary incontinence in Scottish fitness instructors was 28.2% (Stephen et al., 2018). The most frequent form of urinary incontinence in women is stress urinary incontinence (Bø, 2004) and the highest prevalence is found in sports involving high impact (Bø, 2004; Dias et al., 2017). This problem is socially embarrassing and may cause withdrawal from social situations and reduced QoL (Bø, 2004). The high impact (Weintraub, 1994; Palma et al., 2009) and the level of noise (Weintraub, 1994; Palma et al., 2009; Beach and Nie, 2014; Wolniakowska et al., 2021) have also been related/associated with hearing problems and/or hearing loss in FPs. In recent research (Overgaard et al., 2021), hearing loss appears to be closely related to poor QoL. Vocal problems have also been a concern among FPs of GCs (Rumbach, 2013; Rumbach et al., 2015; Dallaston and Rumbach, 2016; Fontan et al., 2017; Aiken and Rumbach, 2018; Estes et al., 2020; Philip et al., 2021; Venkatraman et al., 2021). Research in Australia (Rumbach et al., 2015) shows that approximately 39% of the FPs of GCs reported chronic hoarseness. According to Naqvi and Gupta (2022), partial or total loss of voice may lead to several implications on QoL, since it represents an important instrument for human communication and social interaction. In addition to all these issues, there is a need to address mental health problems among FPs. Mathisen et al. (2021) found a high frequency of sexual harassment experiences and significantly higher scores in symptoms of depression, anxiety and eating disorders among woman that reported it. The prevalence of compulsive exercise and eating disorders in FPs is higher than in the general adult population (Gjestvang et al., 2021). Fitness instructors reported that the body appearance pressure affects them negatively and this may put them at risk of impaired mental health (Mathisen et al., 2020). Most of these professionals believe that their employers give real importance to body appearance (Mathisen et al., 2020).

QoL has been a concern in many populations and FPs should be no exception. The information presented above identifies aspects that may affect the QoL of FPs. Thus, it has been a growing concern in this sector. Despite having an important role in health promotion, the growth/recovery of the fitness sector cannot be achieved in a sustainable way without considering the QoL of its professionals.

Therefore, the aim of this study is to assess the QoL of FPs in Portugal. The objectives are to analyze and compare the QoL of FPs indices concerning gender, marital status, professional title and educational qualification groups, and analyze and correlate the QoL of FPs’ indices with age, number of children, professional experience (years), number of GC/week, number of cardio GC/week, number of strength GC/week, number of mixed GC/week, number of body&mind GC/week, maximal number of GCs per day (GC/day), number of paid working hours/week, number of unpaid working hours/week, monthly net salary, and annual expenses related to the profession.

## Materials and methods

### Participants

The inclusion criterion for this study was: working in the Portuguese fitness industry as a FP with a valid title of TEF or DT. One of the specific criterion to be able to answer the questions concerned with the fitness group class variables was to be performing the function of group class instruction. All the participants that did not complete the QoL questionnaire were excluded from the study. The sample of this study is a convenience sample since the questionnaire was not disseminated to the entire population of FPs working in the Portuguese fitness industry. Of the participants who answered the questionnaire, 388 were considered eligible for this study. Two hundred and ninety-nine individuals answered the questions related to fitness GCs. Regarding socio-demographic characteristics (Table 1), 51.7% of the participants are female and 48.3% are male. The average age is 30.58 ± 7.60 years and the average number of children is 0.34 ± 0.79. Concerning marital status, 66.0% of the participants are single, 16.8% are married, 13.4% are in a non-marital partnership and 3.9% are divorced. Most of the professionals have a bachelor’s degree (69.6%), followed by 18.0% with a master’s degree or higher and 12.4% with a high school level or lower. The participants of this study have an average of 6.96 ± 6.75 years of professional experience and include 56.2% TEFs and 43.8% DTs. The average paid working hours/week are 39.45 ± 19.67 and the unpaid working hours/week are an average of 9.12 ± 7.48. Considering the net salary, 10.3% of the participants earn less than €421,31 per month, 15.6% between €421,32 and €631,97, 25.2% between €631,98 and €842,63, 18.8% between €842,64 and €1.053,29, 12.7% between €1.053,30 and €1.263,95, 7.4% between €1.263,96 and €1.685,27, 5.8% between €1.685,28 and €2.106,59, and 4.0% between €2.106,60 and €2.527,91. Regarding the annual expenses related to the profession, 26.3% spent less than €1.001, 25.8% between €1.001 and €2.100, 22.9% between €2.101 and €3.287, 5 and 25.0% more than €3.287, 5. Concerning the variables of fitness GCs, the average number of fitness GC/week is 9.19 ± 6.85, whereas per typology cardio GC/week is an average of 2.92 ± 4.09, strength GC/week is 2.23 ± 3.03, mixed GC/week is 2.73 ± 5.03 and body and mind GC/week is 1.31 ± 2.42. On average, the GCs FPs instruct a maximum of 3.60 ± 2.11 of GC/day.

**TABLE 1:** Descriptive statistics of the variables.

	Frequency (%)
**Gender**	
Female	51.7
Male	48.3
**Marital status**	
Single	66.0
Married	16.8
Divorced	3.9
Non-marital partnership	13.4
**Educational qualifications**	
High school or level lower	12.4
Bachelor’s degree	69.6
Master’s degree or higher	18.0
**Professional title**	
TEF	56.2
DT	43.8
**Net salary**	
< 421,31€	10.3
421,32 to 631,97€	15.6
631,98 to 842,63€	25.2
842,64 to 1.053,29€	18.8
1.053,30 to 1.263,95€	12.7
1.263,96 to 1.685,27€	7.4
1.685,28 to 2.106,59€	5.8
> 2.106,59€	4.0
**Annual expenses related to the profession**	
< 1.001€	26.3
1.001 to 2.100€	25.8
2.101 to 3.287,5€	22.9
> 3.287,5€	25.0

	***M* ± *SD***

Age	30.58 ± 7.60
Number of children	0.34 ± 0.79
Professional experience	6.96 ± 6.75
GC/week	9.19 ± 6.85
Cardio GC/week	2.92 ± 4.09
Strength GC/week	2.23 ± 3.03
Mixed GC/week	2.73 ± 5.03
Body&Mind GC/week	1.31 ± 2.42
Maximal GC/day	3.60 ± 2.11
Paid working hours/week	39.45 ± 19.67
Unpaid working hours/week	9.12 ± 7.48

### Instruments and procedures

The Portuguese version of the WHOQOL-Bref questionnaire was used (Vaz Serra et al., 2006) to measure the QoL of FPs in Portugal. The WHOQOL-Bref arises from the need for an assessment tool that measures the QoL, which is easy to apply and quick to complete, providing a valid and reliable alternative to the large version of the WHOQoL-100 (Harper et al., 1998; Vaz Serra et al., 2006). This questionnaire consists of 26 items designed to assess four domains related to QoL, physical, psychological, social relations, and environment as well as one facet of OQoLGH (Harper et al., 1998). OQoLGH facet comprises only 2 items, the OQoLGH, and the remaining 24 items are distributed throughout the domains. The physical domain comprises 7 items: pain, energy, sleep, mobility, activities, medication, and work. The psychological domain covers 6 items: positive feelings, thinking, self-esteem, body, negative feelings, and spirituality. The social relations domain includes 3 items: relationships, support and sex. Finally, the environment domain contains the remainder 8 items: safety, home, finances, services, information, leisure, environment, and transport (Harper et al., 1998). Each item is classified by the respondents using a 5-point Likert interval scale of intensity, capacity, frequency, and evaluation (Skevington et al., 2004). The result scores for OQoLGH and the four domains were transformed into an 100-point scale. A questionnaire of sociodemographic and work-related variables of FPs intervention, developed and validated for this study, was also applied (Ramos et al., 2021b). The information was collected between November 2019 and March 2020. To avoid the data not being accurate because of the changes caused by the isolation decreed in Portugal in March, in response to the presence of coronavirus disease (COVID-19), the collection of data was finished before what was initially stipulated. The questionnaires were available on SurveyMonkey, an online platform, and disseminated through social networks, fitness sector associations, higher education institutions and training providers as well as at fitness events and conventions.

### Statistical analysis

The SPSS program version 27.0.1 was used for statistical data treatment. Descriptive statistics, using central tendency (mean) and dispersion (standard deviation) measures, were applied to the OQoLGH and to all domains of QoL, previously converted to a 100-point scale. These statistical techniques were also applied to quantitative characterization variables (age, number of children, professional experience, GC/week, cardio GC/week, strength GC/week, mixed GC/week, body&mind GC/week, maximal GC/day, paid working hours/week, and unpaid working hours/week). In the descriptive analysis of the remaining characterization variables (gender, marital status, educational qualifications, professional title, net salary, and annual expenses related to the profession), a frequency analysis was used. Normal distribution was assumed for all the variables that had, at least, 30 individuals per group, based on the Central Limit Theorem (Pestana and Gageiro, 2020). Regarding group comparison, the *T*-test was applied on dichotomic variables and ANOVA was used when in the presence of more than two groups. The resource for Kruskal-Wallis non-parametric statistic test was needed for the variable with three or more groups, with less than 30 individuals, and without a normal distribution. ANOVA was complemented with Tukey’s or Games-Howell *post hoc* (if the variances were found to be homogenous, or not homogenous, respectively, according to Levene’s test) (Pestana and Gageiro, 2020). The Bonferroni test was the *post hoc* used with Kruskal-Wallis. The effect size was calculated for all the comparisons. To correlate QoL scores with other variables, Pearson’s correlation coefficient was used for scale variables and Spearman association for ordinal variables (net salary and annual expenses related to the profession). The level of significance adopted was *p* < 0.05.

## Results

The results of QoL of FPs (Table 2) reveal the highest indices in the physical domain, with a mean of 74.56 ± 15.18, followed by psychological and social relations domains (72.67 ± 14.22 and 72.23 ± 18.63, respectively). The OQoLGH had a mean of 71.07 ± 16.64 and the lower indices were verified in the environment domain (65.58 ± 14.63).

**TABLE 2:** Descriptive statistic of QoL.

	*M* ± *SD*
OQoLGH	71.07 ± 16.64
Physical	74.56 ± 15.18
Psychological	72.67 ± 14.22
Social relations	72.23 ± 18.63
Environment	65.58 ± 14.63

Concerning the comparison between groups (Table 3), significant differences were observed in physical, psychological, and environment domains on three different variables: gender, marital status, and educational qualifications. In gender, males have average scores higher than females in all domains, including OQoLGH. The differences in marital status were verified between married and divorced groups in the three domains identified above, between married and single groups in the psychological domain and between single and divorced groups in the environment domain. The highest mean scores in all domains, including OQoLGH, belong to the married group and the lowest to the divorced. All the significant differences reported above revealed a low effect size. When it comes to educational qualifications, there were significant differences between master’s degree or higher group and bachelor’s degree group regarding physical and psychological domains, and master’s degree or higher group and high school level or lower group concerning the environment domain. The group with a master’s degree or higher always reveals the best average scores. The significant differences in this variable have a very low effect size. No differences were found between groups according to the professional title. The DTs averages are very similar to the TEFs group, however, DTs show the greatest QoL indices average in almost all domains. The QoL average of the environment domain was the lowest in all groups, regardless of the variable (gender, marital status, educational qualifications, or professional title).

**TABLE 3:** Comparative statistics of QoL between groups.

	OQoLGH		Physical		Psychological		Social relations		Environment	
	M ± SD	*T*-TEST EFFECT SIZE	M ± SD	*T*-test Effect size	M ± SD	*T*-test Effect size	M ± SD	*T*-test Effect size	M ± SD	*T*-TEST Effect size
**Gender**										
Female	69.72 ± 15.71	0.119–0.160	72.63 ± 14.69	0.005*–0.289	70.25 ± 13.56	<0.001*–0.364	71.90 ± 17.57	0.651–0.046	63.74 ± 13.61	0.011*–0.262
Male	72.38 ± 17.60		76.96 ± 15.28		75.36 ± 14.54		72.76 ± 19.67		67.56 ± 15.50	
**Professional title**										
TEF	70.47 ± 17.11	0.442 –0.082	74.39 ± 15.50	0.809–0.025	73.03 ± 14.62	0.571 0.058	71.83 ± 19.98	0.623–0.049	65.07 ± 14.58	0.437–0.080
DT	71.84 ± 16.04		74.77 ± 14.79		72.21 ± 13.72		72.75 ± 16.78		66.23 ± 14.71	

	**M ± SD**	**ANOVA** **effect size**	**M ± SD**	**ANOVA** **effect size**	**M ± SD**	**ANOVA** **effect size**	**M ± SD**	**ANOVA** **effect size**	**M ± SD**	**ANOVA** **effect size**

**Educational qualifications**										
High school level or lower	67.97 ± 17.85	0.055 0.015	74.55 ± 15.57	0.033*^a^ 0.18	72.74 ± 15.35	0.024*^a^ 0.019	71.18 ± 22.01	0.612 0.003	61.91 ± 14.72	0.017*^b^ 0.21
Bachelor’s degree	70.60 ± 17.00		73.47 ± 15.48		71.59 ± 14.50		71.91 ± 18.59		65.22 ± 14.55	
Master’s degree or higher	75.00 ± 13.63		78.78 ± 13.04		76.79 ± 11.52		74.17 ± 16.25		69.46 ± 14.25	

	**M ± SD**	**Kruskal-Wallis** **effect size**	**M ± SD**	**Kruskal-Wallis** **effect size**	**M ± SD**	**Kruskal-Wallis** **effect size**	**M ± SD**	**Kruskal-Wallis** **effect size**	**M ± SD**	**Kruskal-Wallis** **effect size**

**Marital status**										
Single	70.70 ± 16.34	0.143 0.159	74.96 ± 14.56	0.044*^c^ 0.232	71.37 ± 13.99	0.003*^c,d^ 0.339	72.82 ± 18.56	0.110 0.179	65.28 ± 14.81	0.002*^c,e^ 0.354
Married	74.81 ± 15.86		76.37 ± 15.58		77.88 ± 13.99		75.77 ± 14.03		69.95 ± 13.39	
Divorced	64.17 ± 18.22		63.81 ± 16.74		67.22 ± 16.81		60.56 ± 23.03		55.42 ± 10.19	
Non-marital partnership	70.19 ± 18.05		73.42 ± 16.30		74.12 ± 13.34		68.27 ± 21.07		64.48 ± 14.78	

*Significant differences for p < 0.05.

^a^Differences between Bachelor’s degree/Master’s degree or higher.

^b^Differences between High school level or lower/Master’s degree or higher.

^c^Differences between Married/Divorced.

^d^Differences between Single/Married.

^e^Differences between Single/Divorced.

Regarding the correlation and association results (Table 4), unpaid working hours/week is the only variable that established a significant correlation with all domains, including OQoLGH. The level of correlation was considered very low with OQoLGH, physical and social relations domains, and was considered low with psychological and environment domains. This is the only variable with low effect size results. All the other significant correlations/associations that were found, indicated a very low effect size. It is important to emphasize that all correlations with this variable were negative, that is, as the number of unpaid working hours/week increases, the QoL indices decrease. This negative correlation/association trend in all domains and OQoLGH can also be observed with other variables (body&mind GC/week; maximal GC/day; paid working hours/week and annual expenses related to the profession). However, not all of them reveal the existence of a significant correlation/association. The opposite (positive correlation/association trend in all domains and OQoLGH) can be observed with age, professional experience and net salary variables.

**TABLE 4:** Correlation/association statistics of QoL with the variables.

	OQoLGH		Physical		Psychological		Social relations		Environment	
	*R*	*p*	*R*	*p*	*R*	*p*	*R*	*p*	*R*	*p*
Age	0.113	0.026*	0.092	0.070	0.186	0.000*	0.016	0.750	0.157	0.002*
Number of children	0.104	0.041*	0.028	0.584	0.139	0.006*	–0.027	0.596	0.087	0.088
Professional Experience (years)	0.106	0.037*	0.102	0.044*	0.181	< 0.001*	0.005	0.920	0.147	0.004*
GC/week	0.016	0.779	–0.071	0.222	0.029	0.620	–0.001	0.984	–0.022	0.708
Cardio GC/week	0.029	0.621	–0.040	0.488	0.052	0.373	0.045	0.438	0.024	0.681
Strength GC/week	0.037	0.521	–0.010	0.864	0.059	0.308	–0.003	0.953	–0.046	0.432
Mixed GC/week	0.010	0.869	0.001	0.986	–0.008	0.894	0.004	0.944	0.023	0.695
Body&Mind GC/week	–0.069	0.235	–0.122	0.035*	–0.064	0.272	–0.083	0.151	–0.092	0.113
Maximal GFC/day	–0.042	0.472	–0.156	0.007*	–0.009	0.878	–0.046	0.425	–0.136	0.019*
Paid working hours/week	–0.089	0.083	–0.019	0.052	–0.069	0.176	–0.062	0.226	–0.089	0.083
Unpaid working hours/week	–0.143	0.007*	–0.180	< 0.001*	–0.203	< 0.001*	–0.106	0.044*	–0.235	< 0.001*

	**| R|**	***p***	**| R|**	***p***	**| R|**	***p***	**| R|**	***p***	**| R|**	***p***

Net salary	0.113	0.029*	0.087	0.093	0.145	0.005*	0.040	0.434	0.141	0.006*
Annual expenses related to profession	–0.063	0.213	–0.103	0.043*	–0.071	0.164	–0.060	0.240	–0.104	0.041*

*Significant association for p < 0.05. R, Pearson’s correlation coefficient (r). | R|, Spearman’s Rho.

The professional experience variable also reveals many significant correlations with the QoL of FPs. Positive correlations can be observed with OQoLGH, physical, psychological, and environment domains. Age and net salary variables reveal both significant correlations/associations, with positive correlations/associations with OQoLGH, psychological and environment domains. The number of children variable reveals a positive correlation with OQoLGH and with the psychological domain. Maximal GC/day and annual expenses related to the profession indicate both a negative correlation/association with physical and environment domains, and a negative correlation was also found in body&mind GC/week and the physical domain. There were not found any significant correlations between QoL and total of GC/week, cardio GC/week, strength GC/week, mixed GC/week, and paid working hours/week.

## Discussion

The mean indices of QoL suggest that FPs in Portugal are not far from the maximum QoL index (100), considering the scores of OQoLGH (71.07/100), physical (74.56/100), psychological (72.67/100), and social relations (72.23/100) domains. However, taking into consideration the high standard deviation results, it can be concluded that some of the FPs in Portugal are very close to the maximum QoL index, while others are still too far away from it. The mean indices of the environment domain stand out from the results of OQoLGH, physical, psychological, and social domains by more than five points, revealing the lowest indices. The environment is the domain with mean indices results of QoL (65.58/100) farther from the maximum QoL index. The high standard deviation results of QoL in this domain could represent a concern, once these results suggest that some of the FPs in Portugal have indices of QoL in the environment domain that are too close to the middle of QoL index (50). The fact that the environment domain has the lowest QoL indices is coincident with the results of other studies of FPs (Bevilacqua et al., 2014), weight training FPs (Simões et al., 2011) and Crosstaining FPs (Neto et al., 2020) conducted in Brazil. These findings are also in line with the results of job satisfaction of FPs in Portugal (Ramos et al., 2021a), as the environment is the domain that covers the factors reported with the lowest level of satisfaction (salary and opportunities for promotion). In the Simões et al. (2011) study, the results of OQoLGH and the four domains have the same order as the results in the present study. Compared to other Studies of QoL in FPs (Simões et al., 2011; Bevilacqua et al., 2014; Neto et al., 2020), we can observe, in all of them, lower standard deviations than in the present study and higher mean indices of OQoLGH and all domains. In the OQoLGH, the differences in the mean indices are 7–17 points higher, 6/7–9/10 points higher in the physical, psychological and social relations domains and 3–10 points higher in the environment domain. According to Ferreira and Santana (2003), the Portuguese norms regarding the perception of the state of health and QoL of the working population are similar to the results of the present study but lower than the American, Italian and French norms. Monteiro (2020) affirms that the Portuguese population has shown little QoL in recent years and the results of this study, especially when compared to the results of other countries, seem to support this idea.

The indices of QoL (OQoLGH and the four domains) in this study were 5–8 points lower than the pilot study of QoL of FPs in Portugal (Vieira et al., 2019). The low percentage of individuals with a master’s degree or higher and the low average of years of professional experience (both verified with the participants in this study) are possible explanations for these results, since a higher level of education reveals better average scores in OQoLGH and in all domains, and the professional experience was positively correlated with QoL (with four significant differences in five possible). Similar results were found in other studies (although not with the same target population). Purba et al. (2021) suggest that higher education levels indicate better QoL in almost all domains (except the domain of the social relation) and according to Molsted et al. (2021), education levels reveal a positive relation with physical and mental component scales. The results of job satisfaction in FPs in Portugal (Ramos et al., 2021a) reveal slightly higher values of satisfaction in 12 of the 16 factors analyzed in the higher qualifications group. Regarding professional experience, higher levels of job satisfaction of FPs in Portugal were revealed by the group with more years of professional experience (10 or more), with significant differences in 14 of the 16 factors (Ramos et al., 2021a). Bratland-Sanda et al. (2015) found that working years as a fitness instructor were one of the factors positively associated with injury, but these findings do not appear to be significant for the perception of the QoL physical domain of FPs in Portugal.

In line with professional experience, the age variable had also revealed a positive correlation with QoL (OQoLGH and all domains), although the significant correlation occurs only with OQoLGH, psychological and environment domains. These results are also consistent with Ramos et al. (2021a) findings on FPs job satisfaction levels. In that research, older FPs (45–65-year-old group) were more satisfied in all of the 16 factors that were analyzed, being significantly higher in 10 of them. More maturity to deal with the psychological challenges of the profession could be one of the possible justifications for the positive relation of age with the psychological domain. However, studies about QoL of FPs that can support this theory were not found.

Despite these results, the positive correlations between QoL and the variables age and professional experience could be arguable. The average of these two variables is something to consider, once the participants in this study have an average of 6.69 ± 6.75 years of professional experience and 30.58 ± 7.59 years of age. According to Instituto Português do Desporto e Juventude [IPDJ] (2022), the average age of FPs with valid titles in Portugal in 2021 was 37 years, 67% being 40 years old or less and only 7% being more than 50 years old. Considering the retirement age of 66 in Portugal (Portaria n.° 307/2021), those averages correspond practically to the beginning of a career. It is also important to consider the number of FPs that have not validated their professional title in the last years. From 2010 to 2021, 33,359 professional titles were issued in Portugal (26,361 TEFs and 6,998 DTs), but only 63% of the TEFs (16,604) and 76% of the DTs (5,342) titles remained valid in 2021 (Instituto Português do Desporto e Juventude [IPDJ], 2022). Therefore, these results could be a consequence of the natural selection of the fitness industry, which clearly increases the need to understand whether the QoL of FPs improves with age and years of professional experience or if only the FPs with better levels of QoL remain in the profession.

The differences between gender found in the present research suggest that female FPs have a lower QoL than male FPs. The significant differences found in the physical, psychological, and environment domains may be related to the problems identified in some studies. Despite the lack of differences in job satisfaction between gender FPs in Portugal (Ramos et al., 2021a), a Brazilian study (Anversa et al., 2019) reveals that female FPs had lower levels of satisfaction than male FPs in some physiological needs and regarding their safety at work. Mathisen et al. (2021) found frequencies of 30% of sexual harassment among women FPs at the workplace (higher than men frequencies). Having experienced sexual harassment had significantly higher scores in symptoms of depression, anxiety and eating disorders compared to women with no such experience. Another issue reported as a common problem in the female population is urinary incontinence (Bø, 2004). Bø (2004) suggests that this is a problem that can reduce QoL because it is socially embarrassing and may cause withdrawal from social situations. In a study of QoL of young industry workers, Louzado et al. (2021) suggest double working hours for women (employment and home activities) as a possible justification for differences found between gender (lower indices of QoL for females).

Marital status seems to be an important characteristic of QoL. Being divorced was the characteristic that showed the lowest mean indices of QoL levels (OQoLGH and four domains) of all groups (considering the variables: gender, professional title, marital status, and educational qualifications) and being married showed one of the highest mean indices (once this position was shared with an educational qualification characteristic, that is having a master’s degree or higher, both with very similar averages). The significant differences between these groups (married and divorced) in physical, psychological, and environment domains may be explained by the support that a partner can give in difficult situations (Molsted et al., 2021) and the possibility to share daily basis activities and worries.

Most of our respondents were single (66.0%) and with no children (once the mean and standard deviation of this variable was 0.34 ± 0.79). These characteristics seem to have a negative impact on the psychological domain of the participants in this study, since being single was significantly different (with lower indices) from being married and the number of children showed a significant positive correlation with the psychological domain. No studies were found with FPs population that can support these results.

Concerning the professional title, no significant differences between TEFs and DTs were found. These two groups revealed different opinions on job satisfaction in the factors “opportunities for promotion” and “job stability” (Ramos et al., 2021a). Therefore, differences between these groups and QoL psychological or environment domains were expected. However, the absence of significant differences is not entirely a surprise, because the functions are not completely distinct (Lei n.° 39/2012), since DT title also covers the functions of TEF. It is also possible that some of the DTs that participated in this study do not even assume coordination functions or technical direction. On the other hand, although TEFs cannot assume the function of technical direction, they may be responsible for coordinating certain areas in the gym. Consequently, it is possible that the professional title does not correspond to a clear division of the functions performed on the field by the FPs in Portugal and this could be the reason behind the absence of differences in QoL between TEFs and DTs. The lack of results in the comparison of these two groups reinforces the need to review the existing legislation for FPs, a subject already addressed by Franco (2020).

*“It is expected that the legislation that regulates fitness professionals will be adapted, particularly in terms of levels and extension of contexts, so that professionals can be differentiated through the specificity, complexity and depth of their skills and knowledge, in the scope of their intervention, valuing the education and regulation of the sector (*…*)”* (Franco, 2020, 7).

The biggest surprise of the results is the lack of significant relations between the number of GC/week and QoL and the same happens to cardio, strength, and mixed GC/week. Considering the number of studies that reveal the high prevalence of musculoskeletal problems in the FPs related/associated with GC (du Toit and Smith, 2001; Bø, 2004; Bratland-Sanda et al., 2015; Domene et al., 2017), significant correlation results between these variables and QoL physical domain were expected. According to Bratland-Sanda et al. (2015), the prevalence of acute, overuse and both acute and overuse injuries in FPs GCs in Norway were always higher in the group with more GC per week (GC/week). This group has an average of 7.5 h/week of GP and the results of the present study reveal an average of 9.19 ± 6.85 GC/week. A significant relation result was also expected in the social relation’s domain, once the prevalence of voice (Rumbach, 2013; Rumbach et al., 2015; Dallaston and Rumbach, 2016; Fontan et al., 2017; Estes et al., 2020; Philip et al., 2021; Venkatraman et al., 2021) and hearing problems (Weintraub, 1994; Palma et al., 2009; Beach and Nie, 2014; Wolniakowska et al., 2021) was related/associated in other studies with instruction of GC and possible communication difficulties could result in limitations of QoL levels in this domain. Urinary incontinence is also a problem related/associated with impact GC. Some authors suggest that this can have a negative influence on the QoL (Bø, 2004; Stephen et al., 2018).

However, maximal GC/day variables reveal a negative correlation with QoL (OQoLGH and all domains) and this relation was significant in physical and environment domains. It seems that maximal GP/day has a bigger impact on QoL than de number of GC/week. Considering the mean of both variables (3.6 ± 2.11 maximal GP/day and 9.19 ± 6.84 GP/week) these results may increase the importance of respecting the training recovery. According to Bishop et al. (2008), different training stresses require different lengths of recovery. Therefore, the level of fatigue in the workout will change according to the recovery needs. Full training recovery is essential for athletes to achieve optimal performance and improvement (Bishop et al., 2008), and while improved performance is not a key goal for a fitness GC instructor, training volume may be similar in some cases. There is a need to understand that the training volume of a FP does not correspond to the number of classes he teaches. There may also be training for class preparation and non-occupational physical activity, which appears to improve the stress levels of fitness instructors (Marín-Farrona et al., 2021). This has been becoming very usual in instructors despite the volume of physical activity related to work.

Contrary to what was expected, body&mind GC/week was negatively related with the physical domain. Once more, the high prevalence of musculoskeletal problems in the FPs is usually related/associated with high impact, intense or repetitive movements in GC (Garrick et al., 1986; du Toit and Smith, 2001; Bø, 2004; Bratland-Sanda et al., 2015; Domene et al., 2017; Stephen et al., 2018). Although this is the only significant correlation found, this variable exposes a tendency of a negative relation with QoL (OQoLGH and all domains), which is questionable, especially concerning the psychological domain. Assuming that lower levels of QoL, especially in the physical domain, are a consequence of a higher number of body&mind GP/week could be a mistake because it is also possible that FPs with lower indices of QoL in the physical domain direct their intervention to the instruction of GC with less mechanical loading, like body&mind GC. Therefore, these results could be a sign of FPs’ adaptation to the profession so that they are able to remain in the fitness industry. More research is needed to better understand these findings.

Concerning the net salary and paid working hours/week variables, similar results were expected if working more hours corresponded to earning more money, but this does not happen with the participants in this study. The net salary has significant positive associations with OQoLGH, psychological and environment domains and paid working hours/week does not reveal any significant relation with QoL. In fact, more net salary indicates a positive association tendency with QoL (OQoLGH and four domains), whereas paid working hours/week show opposite behavior (negative correlation tendency). Unpaid working hours/week and annual expenses related to the profession also reveal a negative correlation/association tendency with QoL (OQoLGH and four domains). Unpaid working hours were the only variable that had a significant correlation with OQoLGH and all domains. Hence, 51.1% of the participants in this study receive €842, 63 or less, having an average amount of paid working hours/week of 39.45 ± 19.67 and an average of unpaid working hours/week of 9.12 ± 7.48. In addition to these results, 47.9% of the participants spent more than €2.100 per year in expenses related to the profession. These findings are in agreement with the results of lower satisfaction levels concerning salary revealed in studies that address job satisfaction of FPs (Koustelios et al., 2003; Bevilacqua et al., 2014; Bernabé et al., 2017; Ramos et al., 2021a).

A general analysis of QoL indicates that OQoLGH and psychological domain reveal a positive significant correlation with age, number of children and professional experience. A negative significant correlation was established with unpaid working hours/week. The physical domain also reveals five significant correlations/associations, being positively related to professional experience and negatively related/associated with body&mind GC/week, maximal GC/day, unpaid working hours, and annual expenses related to the profession. In the domain of social relations, the lowest number of significant correlations was found, since only unpaid working hours/week reveal a significant negative correlation. In a job satisfaction study on FPs in Portugal (Ramos et al., 2021a), the most satisfied workers also had the best results regarding their work colleagues. The largest number of significant correlations/associations were verified in the environment domain, with six correlations/associations. Age, professional experience and net salary variables indicate a positive relation/association with this domain and maximal GC/day, unpaid working hours and expenses related to the profession variables suggest a negative relation/association.

Five of the variables related to work reveal significant correlations/associations with the QoL of FPs. Despite these results exposing a very low or low effect size and bearing in mind the number of FPs that have not validated their professional title in the last few years (Instituto Português do Desporto e Juventude [IPDJ], 2022), these findings need to be considered in the fitness industry so that the working conditions of the FPs can be improved, hoping that it results in better levels of QoL, fewer FPs abandoning their careers and also leads to better work ability of the FPs.

There is clearly a lack of research on QoL of FPs and many questions remain unanswered. The struggle of the fitness industry due to the COVID-19 pandemic has affected FPs directly. It reflected itself in fewer jobs and a higher number of customers per instructor (Pedragosa and Cardadeio, 2021). Therefore, there is also a possibility that the QoL of FPs has been affected, too. Some studies refer to FPs as an important role model (Franco, 2020, Gjestvang et al., 2021; Reinboth et al., 2022), with a potentially great impact on their clients’ exercise behaviors and attitudes (Reinboth et al., 2022). Considering that FPs can be one of the greatest assets in the recovery of fitness industry, their QoL levels should be crucial, as they are a part of this process and work as role models for fitness practitioners.

## Conclusion

QoL mean results with a high standard deviation highlight the different levels of QoL of FPs in Portugal, with some concerning indices. Mean indices of QoL of the FPs in Portugal are lower than in other countries. The environment is the domain that revealed the lowest scores of QoL. The results reveal that being a male, married and having a master’s degree or higher were the characteristics that indicated better indices of QoL. Age, professional experience and net salary indicate a positive correlation/association with QoL with a few significant results. Body&mind GP/week, maximal GP/day, paid and unpaid working hours/week, and annual expenses related to the profession reveal a negative relation/association with QoL. Paid working hours/week do not reveal any significant correlation with QoL. However, unpaid working hours/week had the highest number of significant correlations with QoL. Maximal GC/day results are a concern for the physical health of FPs, although they could also represent an important finding to help minimize the physical problems among FPs. Body&mind GC/week results could represent FPs adaptation to the job that allows them to remain in the profession. No significant relations between QoL and the number of GP/week, cardio GP/week, strength GP/week, mixed GP/week, and paid working hours/week were found. There is a possibility that the FPs with the lowest indices of QoL have abandoned the profession and the number of significant relations/correlations between QoL and variables related to work suggest the need for the fitness industry contemplate some changes in the FPs working conditions. More research is needed about the QoL of FPs, taking into consideration the remaining doubts. The important role that these professionals can represent in the recovery of the fitness industry, the role they can play in the promotion of physical activity to give a better QoL to the practitioners and for a healthier society, the interest in improving the work conditions and the ability to work (related to QoL) and in minimizing the number of FPs abandoning the profession, represents an encouragement for the continuance of research on this topic.

## Data availability statement

The raw data supporting the conclusions of this article will be made available by the authors, without undue reservation.

## Ethics statement

The studies involving human participants were reviewed and approved by the ethics and scientific board of the Polytechnic Institute of Santarém, n.° 8A-2022 ESDRM. The patients/participants provided their written informed consent to participate in this study.

## Author contributions

IV wrote the sections of the manuscript. All authors have contributed to the conception and design of the study, revision of the manuscript, read, and approved the submitted version.

## Footnotes

1 At the time of the questionnaire the minimum salary in Portugal was around €700.
